# Supplementary figures and images for: Interdependence of Molecular Lesions That Drive Uveal Melanoma Metastasis
Source: Int J Mol Sci. 2023 Oct 26;24(21):15602. doi: 10.3390/ijms242115602 (PMC10648765; doi:10.3390/ijms242115602)

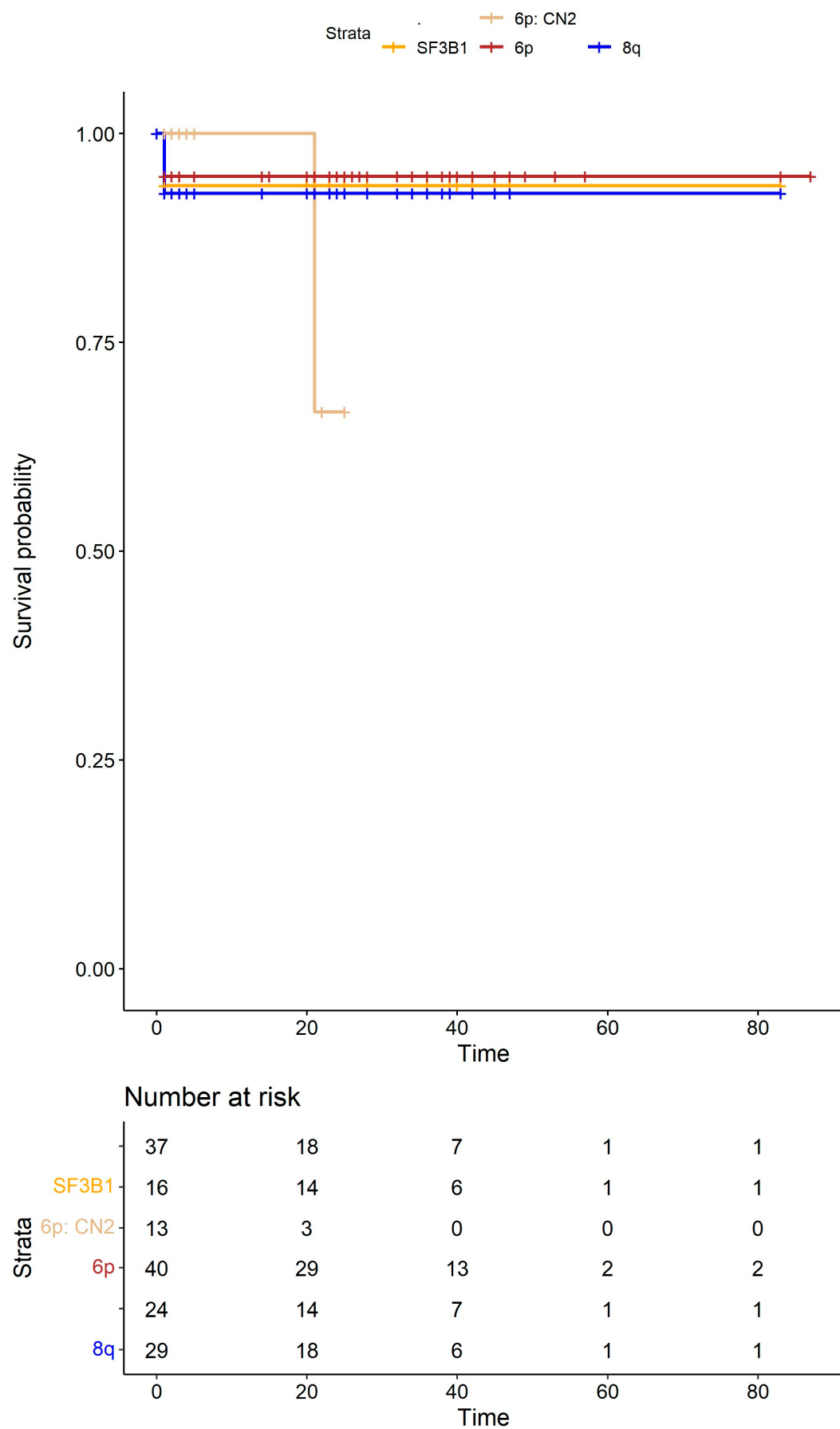

**Figure S1:** Survival curves of genomic factors in disomic patients.

Supplement: Supplementary file 1 [file ijms-24-15602-s001.zip › ijms-2662776-Supplementary Figure S1.pdf]
